# *Dermacentor occidentalis* Ticks and Link to *Rickettsia lanei* Infections, California, USA

**DOI:** 10.3201/eid3201.251261

**Published:** 2026-01

**Authors:** Will S. Probert, Chantha Kath, Naomi Putirka, Megan E.M. Saunders, Brenda Bermudez, Matthew I. Cazas, Alex Espinosa, Hannah Romo, Jill K. Hacker

**Affiliations:** Author affiliations: California Department of Public Health, Richmond, California, USA (W.S. Probert, C. Kath, M.E.M Saunders, B. Bermudez, M.I. Cazas, A. Espinosa, H. Romo, J.K. Hacker); San Francisco State University, San Francisco, California, USA (N. Putirka)

**Keywords:** spotted fever group rickettsiosis, Rocky Mountain spotted fever, bacteria, vector-borne infections, ticks, real-time PCR, whole-genome sequencing, California, United States

## Abstract

*Rickettsia lanei* is a newly recognized spotted fever group rickettsial species that causes severe Rocky Mountain spotted fever–like illness. We used genome sequencing, enabled by hybridization capture-based target enrichment, to establish *Dermacentor occidentalis* ticks as the likely source of a human infection with *R. lanei* in California, USA.

Spotted fever group (SFG) rickettsioses are acute undifferentiated febrile illnesses caused by tickborne transmission of intracellular gram-negative bacteria belonging to SFG *Rickettsia*. Rocky Mountain spotted fever (RMSF) is caused by *R. rickettsii* subspecies *rickettsii* and Pacific Coast tick fever by *R. rickettsii* subsp. *californica*; those 2 SFG rickettsioses are the most frequently reported in California, USA (*1*,*2*). We recently described 2 cases, 1 with disease onset in 2023 and 1 identified retrospectively with onset in 2004, of severe RMSF-like illness in northern California caused by a newly recognized SFG rickettsial pathogen, *Rickettsia* sp. CA6269 (*3*). This novel rickettsial genotype was first identified in *Haemaphysalis leporispalustris* ticks in Sonoma County, California, and, more recently in *H. leporispalustris* ticks in Maine, USA (*4*,*5*). Subsequently, whole-genome sequencing of rickettsial strain HLP 7421, isolated in 1961 from a pool of *H. leporispalustris* ticks collected in Montana, USA, supported classification of the *Rickettsia* sp. CA6269 genotype as a new species, *R. lanei* (*6*). In this study, we report on the detection of *R. lanei* in *Dermacentor occidentalis* and *H. leporispalustris* ticks collected at or near locations of exposure for the 2004 and 2023 cases.

## The Study

The California Department of Public Health monitors prevalence of SFG *Rickettsia* annually by collecting and testing *Dermacentor* spp. ticks (*3*,*7*). In 2024, SFG *Rickettsia* surveillance was enhanced to include collection of *H. leporispalustris* and additional *Dermacentor* spp. ticks from areas associated with exposures for the 2004 (Marin and San Mateo Counties) and 2023 (Alameda and Contra Costa Counties) cases. In addition, *H. leporispalustris* ticks were collected from an area in Sonoma County where *R. lanei*–positive ticks were originally described (*4*). In 2024, a total of 3,607 adult and nymphal ticks were tested for SFG *Rickettsia*: 2,872 *D. occidentalis* ticks collected from 34 of the 58 California counties and 69 *D. similis* and 666 *H. leporispalustris* ticks collected from 6 counties (Figure).

**Figure:**
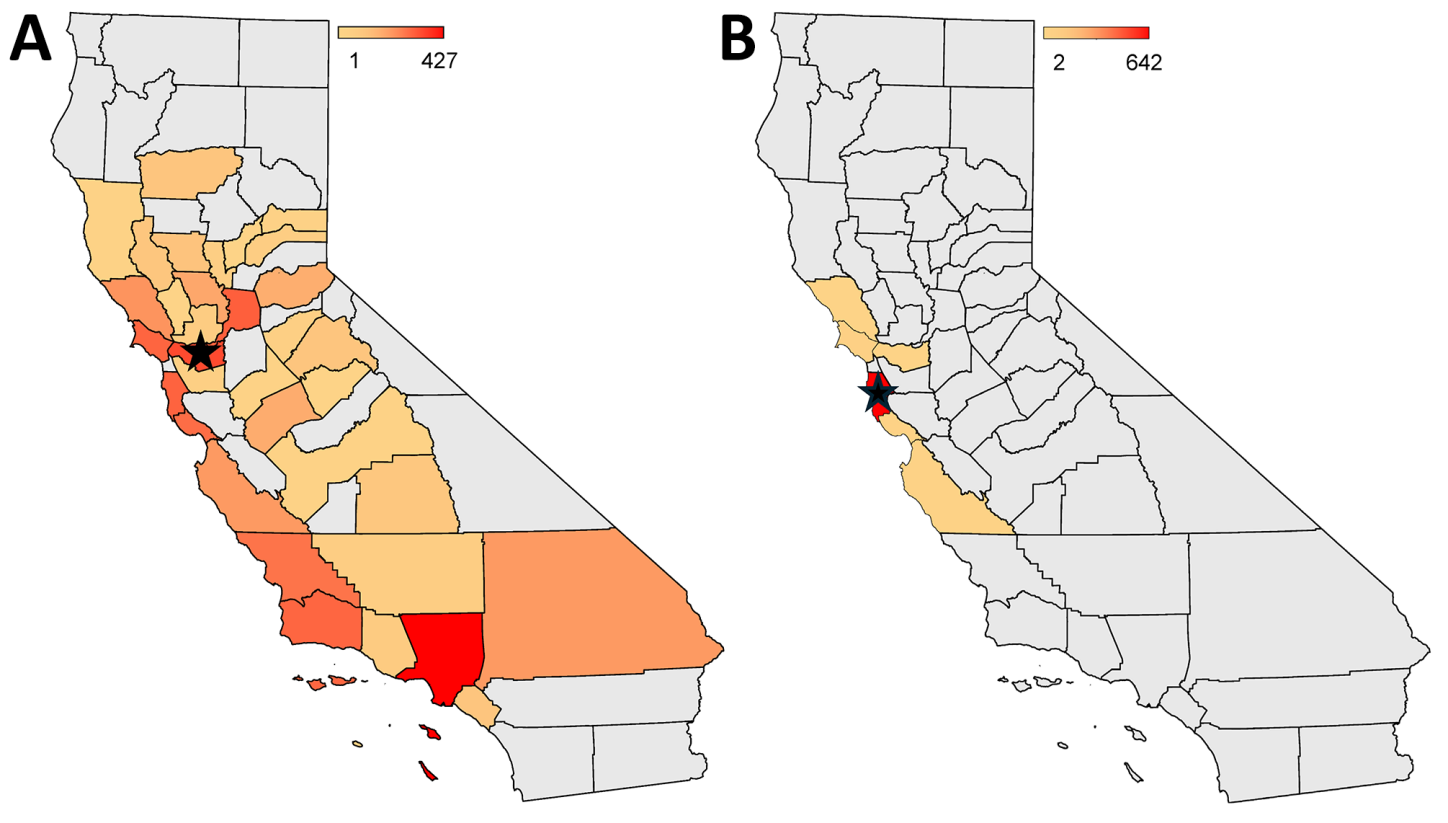
Heat maps displaying the number of *Dermacentor occidentalis* (A) and *Haemaphysalis l*e*porispalustris* (B) ticks tested for spotted fever group *Rickettsia* in 2024, by county, in study of *D. occidentalis* ticks and link to *Rickettsia lanei* infections, California, USA. Color scale represents number of ticks. Black star represents the location of ticks testing positive by PCR for *R. lanei*: 1 *D. occidentalis* tick in Contra Costa County (A) and 2 *H. leporispalustris* ticks in San Mateo County (B). Gray shading indicates counties in which tick collection was not attempted or the tick species was not found.

We extracted and purified tick nucleic acids and screened for *Rickettsia* spp. using PanR8 real-time PCR (rPCR) (*3*,*8*). We tested all positive samples for *R. rickettsii* subsp. *californica* using the *nusG* rPCR and *R. rickettsii* subsp. *rickettsii*/*R. lanei* using RRi6 rPCR (*8*,*9*). We used a fourth rPCR to distinguish *R. lanei* from *R. rickettsii* subsp. *rickettsii* (*3*). We detected *Rickettsia* spp. in 14% of *D. occidentalis* ticks, 20.3% of *D. similis* ticks, and 3.5% of *H. leporispalustris* ticks (Table 1). We detected *R. rickettsii* subsp. *californica* only in *D. occidentalis* ticks (1.3%) and did not detect *R. rickettsii* subsp. *rickettsii* in any ticks. Two *H. leporispalustris* nymphs (24-5179 and 24-6522) collected 34 days apart from the same site in San Mateo County and 1 *D. occidentalis* adult female tick (24-7980) from Contra Costa County were positive for *R. lanei* by rPCR with cycle threshold values of 27.5 (24-5179; DNA diluted 1:20), 27.5 (24-6522; DNA diluted 1:20), and 18.4 (24-7980; DNA neat) (Appendix). The overall prevalence of *R. lanei* was 0.3% in *H. leporispalustris* ticks and 0.03% in *D. occidentalis* ticks (Table 1).

**Table 1:** PCR detection of *Rickettsia* in *Dermacentor* and *Haemaphysalis* ticks collected in California, 2024

Tick species	No. ticks tested	No. PCR-positive ticks (%)			
		*Rickettsia* genus	*R. rickettsii* subspecies *rickettsii*	*R. rickettsii* subsp. *californica*	*R. lanei*
*Dermacentor occidentalis*	2,872	402 (14)	0	38 (1.32)	1 (0.03)
*Dermacentor similis*	69	14 (20.29)	0	0	0
*Haemaphysalis leporispalustris*	666	23 (3.45)	0	0	2 (0.3)

We performed hybridization capture-based target enrichment sequencing to investigate genome relatedness of *R. lanei* strains from the 2023 case (strain CA23RL1) and ticks 24-5179, 24-6522, and 24-7980 (*10*). We amplified remnant nucleic acids from the plasma specimen collected on day 7 from the 2023 case using the REPLI-G whole genome amplification kit (QIAGEN, https://www.qiagen.com). Unfortunately, nucleic acids from the 2004 case had been depleted. We processed tick DNAs without whole-genome amplification. We performed DNA library preparation and hybridization capture-based target enrichment using a customized panel of ≈10,000 120-bp biotinylated oligonucleotides designed to span the genome of *R. rickettsii* subsp. *californica* (GenBank accession no. CP003308.1) end-to-end using the KAPA HyperCap workflow version 3 (Roche, https://www.roche.com). We sequenced enriched libraries on a MiSeq using reagent kit v2 (500 cycles) (Illumina, https://www.illumina.com).

We assembled a near whole-genome sequence of strain CA23RL1 from the 2023 case by mapping sequencing reads to the *R. lanei* type strain HLP 7421 genome (GenBank accession no. CP172233) using Geneious Prime v2022.0.2 (https://www.geneious.com). The percentage of CA23RL1 reads that mapped to HLP 7421 was 29.9%, providing 99.4% genome coverage with an average depth of 400 reads. We mapped unused reads to the genome of *R. rickettsii* subsp. *californica* and inserted the resultant mapped sequences into the CA23RL1 consensus sequence. We used Sanger sequencing to close genome sequence gaps and resolve repetitive regions, establishing a complete genome sequence of 1,270,942 bp for CA23RL1. Genome relatedness was 98.5% between CA23RL1 and *R. rickettsii* subsp. *rickettsii*, 98.7% between CA23RL1 and *R. rickettsii* subsp. *californica*, and 99.7% between CA23RL1 and *R. lanei*, as determined by the orthologous average nucleotide identity algorithm (*11*).

We assembled genome sequences for the tick samples by mapping reads to the CA23RL1 genome. Genome coverage for the tick samples was >99.4%; average depth was >1,100 sequencing reads. We resolved repetitive regions and genome sequence gaps using Sanger sequencing and aligned complete genome sequences for the tick samples to the CA23RL1 genome using Mauve version 1.1.3 to determine genetic relatedness (*12*). The *D. occidentalis* 24-7980 genome sequence was identical to the CA23RL1 genome sequence, whereas those of the *H. leporispalustris* samples (24-5179 and 24-6522) were identical to one another but differed from the CA23RL1 and 24–7980 genome sequences by 19 nt polymorphisms and 4 insertions/deletions (Table 2).

**Table 2:** Comparison of tick genome sequences to *Rickettsia lanei* strain CA23RL1 genome sequence in study of *Dermacentor occidentalis* ticks and link to *Rickettsia lanei* infections, California, USA

Tick identifier	Tick species	County of collection	Genome size, bp	Single-nucleotide polymorphisms	Insertions/deletions
24-5179	*Haemaphysalis leporispalustris*	San Mateo	1,270,870	19	4
24-6522	*H. leporispalustris*	San Mateo	1,270,870	19	4
24-7980	*Dermacentor occidentalis*	Contra Costa	1,270,942	0	0

Sequencing data are available in the National Center for Biotechnology Information BioProject database (accession no. PRJNA1261853). Limitations to our study are the lack of long-read next generation sequencing data to confirm lengthy repetitive DNA regions and the potential bias in using closely related reference sequences for guiding capture probe design and genome assembly.

## Conclusions

We investigated the role of *Dermacentor* spp. and *H. leporispalustris* ticks as reservoirs and potential vectors of *R. lanei* in California and found a very low prevalence of infection. Ticks infected with *R. lanei* were only detected in counties identified as locations of exposure for the 2 cases: 2 *H. leporispalustris* nymphs collected in San Mateo County near a location of exposure for the 2004 case and 1 *D. occidentalis* adult tick collected in Contra Costa County at a site of exposure for the 2023 case. The positive *D. occidentalis* tick was collected at 1 of 5 golf courses visited by the case-patient within 14 days of illness onset (*3*). This ecoepidemiologic association and identical *R. lanei* genomic sequence match between the 2023 case and the *D. occidentalis* tick strongly implicate this tick species as the source of disease transmission. This conclusion is further supported by the observation that *D. occidentalis* ticks infest humans much more frequently than do *H. leporispalustris* ticks (*13*).

The *R. lanei* genome sequence from the *H. leporispalustris* ticks shared a high degree of sequence identity (orthologous average nucleotide identity >99.9%) with the sequence from the 2023 case and the *D. occidentalis* tick. Although it is unlikely that *H. leporispalustris* plays a role in the human transmission of *R. lanei* given both its preference for lagomorphs and the rarity of human infestations, this tick species may serve as a key vector for maintenance of *R. lanei* in nature, much as it does for *R. rickettsii* (*14*,*15*). Future studies are warranted to confirm vector competency of *D. occidentalis* and *H. leporispalustris* for *R. lanei* transmission. Acknowledging the severity of the 2 *R. lanei* infections and the broad distribution of the tick species, our results highlight the role of ecoepidemiologic investigations in identifying risk factors and guiding mitigation strategies for preventing vectorborne diseases.

Appendix Additional information about *Dermacentor occidentalis* ticks and link to *Rickettsia lanei* infections, California, USA.
