# Supplementary material for: Dermacentor occidentalis Ticks and Link to Rickettsia lanei Infections, California, USA
Source: Emerg Infect Dis. 2026 Jan;32(1):122–5. doi: 10.3201/eid3201.251261 (PMC12870014; doi:10.3201/eid3201.251261)
Supplement: Appendix — Additional information about Dermacentor occidentalis ticks and link to Rickettsia lanei infections, California, USA. [file 25-1261-Techapp-s1.pdf]

Article DOI: https://doi.org/10.3201/eid3201.251261

EID cannot ensure accessibility for supplementary materials supplied by authors. Readers who have difficulty accessing supplementary content should contact the authors for assistance.

Dermacentor occidentalis Ticks and Link to *Rickettsia lanei* Infections, California, USA

Appendix

Appendix Table. Number of ticks tested for spotted fever group *Rickettsia* using real-time PCR by California county

	Number of ticks tested by real-time PCR		
California county*	*Dermacentor occidentalis*	*Dermacentor similis*	*Haemaphysalis leporispalustris*
Alameda	8	0	0
Calaveras	8	0	0
Colusa	32	0	0
Contra Costa	276	30	2
El Dorado	75	0	0
Fresno	3	0	0
Kern	13	0	0
Lake	27	0	0
Los Angeles	427	0	0
Marin	223	2	11
Mariposa	1	0	0
Mendocino	3	0	0
Merced	78	0	0
Monterey	115	1	5
Napa	1	0	0
Nevada	13	0	0
Orange	28	0	0
Sacramento	235	0	0
San Bernardino	115	0	0
San Luis Obispo	191	0	0
San Mateo	227	33	642
Santa Barbara	221	0	0
Santa Cruz	205	2	4
Sierra	4	0	0
Solano	14	0	0
Sonoma	128	1	2
Stanislaus	3	0	0
Sutter	10	0	0
Tehama	29	0	0
Tulare	24	0	0
Tuolumne	28	0	0
Ventura	17	0	0
Yolo	89	0	0
Yuba	1	0	0

* Ticks were not tested from the following California counties: Alpine, Amador, Butte, Del Norte, Glenn, Humboldt, Imperial, Inyo, Kings, Lassen, Madera, Modoc, Mono, Placer, Plumas, Riverside, San Benito, San Diego, San Francisco, San Joaquin, Santa Clara, Shasta, Siskiyou, Trinity.
